# Small RNA Sequencing Reveals Differentially Expressed miRNAs in Necrotizing Enterocolitis in Rats

**DOI:** 10.1155/2020/5150869

**Published:** 2020-09-02

**Authors:** Ren-qiang Yu, Min Wang, Shan-yu Jiang, Ying-hui Zhang, Xiao-yu Zhou, Qin Zhou

**Affiliations:** ^1^Department of Neonatology, Children's Hospital of Nanjing Medical University, Nanjing, China; ^2^Department of Neonatology, The Affiliated Wuxi Maternity and Child Health Care Hospital of Nanjing Medical University, Wuxi, China

## Abstract

Necrotizing enterocolitis (NEC) is the leading cause of death due to gastrointestinal disease in preterm infants. The role of miRNAs in NEC is still unknown. The objective of this study was to identify differentially expressed (DE) miRNAs in rats with NEC and analyze their possible roles. In this study, a NEC rat model was established using Sprague-Dawley rat pups. Small RNA sequencing was used to analyze the miRNA expression profiles in the NEC and control rats. Gene Ontology (GO) and Kyoto Encyclopedia of Genes and Genomes (KEGG) analyses were carried out to identify target mRNAs for the DE miRNAs and to explore their potential roles. The DE miRNAs were verified by real-time quantitative PCR (RT-qPCR). The status of intestinal injury and the elevated levels of inflammatory cytokines in the NEC group confirmed that the NEC model was successfully established. The 16 miRNAs were found to be differentially expressed between the NEC group and the control group of rats. Bioinformatics analysis indicated that the parental genes of the DE miRNAs were predominantly implicated in the phosphorylation, cell migration, and protein phosphorylation processes. Moreover, the DE miRNAs were mainly found to be involved in the pathways of axon guidance, endocytosis, and focal adhesion, as well as in the Wnt signaling pathway, which is related to colitis. The expression patterns of the candidate miRNAs (rno-miR-27a-5p and rno-miR-187-3p), as assessed by RT-qPCR, were in accordance with the expression patterns obtained by miRNA-sequencing. The miRNA/mRNA/pathway network revealed that rno-miR-27a-5p and rno-miR-187-3p might be involved in NEC via the Wnt signaling pathway. We found an altered miRNA expression pattern in rats with NEC. We hypothesize that rno-miR-27a-5p and rno-miR-187-3p might mediate the NEC pathophysiological processes via the Wnt signaling pathway.

## 1. Introduction

Necrotizing enterocolitis (NEC) is considered one of the leading causes of neonatal morbidity and mortality [1]. NEC is common in neonates and premature infants, especially in very low birth weight infants, with a death rate of approximately 30% [2, 3]. The major risk factors for the development of NEC are prematurity, bacterial colonization, and management of formula feeds [4]. However, the pathogenesis of NEC consists of multiple factors such as the imbalance of anti-inflammatory and proinflammatory factors and the deterioration of systemic sepsis [5]. It is difficult to diagnose early clinical NEC, given its nonspecific clinical features such as acute phase proteins and cytokines. NEC is also difficult to distinguish from other diseases with similar symptoms [6, 7]. Recently, a systematic large-scale meta-analysis analyzed microRNA (miRNA) expression profiles to screen biomarkers for NEC in newborns [8]. Significant differential expression of miRNAs has been reported between ulcerative colitis and ulcerative colitis-related colorectal cancer in the mouse [9]. However, little is known about the role (and related molecular mechanism) of miRNAs in NEC. Therefore, it is of great importance to screen novel miRNA biomarkers to diagnose NEC.

miRNAs are small noncoding RNAs of 18-24 nucleotide length. Abnormal expression of miRNAs is associated with human cancer and diseases [10]. A variety of miRNAs has been identified as potential biomarkers for prognosis and diagnosis of human cancer and diseases through next-generation sequencing (NGS) technology [11]. Additionally, miRNAs serve as important regulators of mRNA expression due to their ability to bind to argonaute protein [12]. Reports show that abnormal expression patterns of miRNAs contribute to inflammatory response in inflammatory bowel disease [13]. Thus, miRNAs have diagnostic and therapeutic value. A recent study used a microarray to identify plasma miR-1290 as a novel biomarker that can effectively differentiate NEC from other diseases presenting similar symptoms, thus recognizing it as a potential therapeutic target [14]. miRNA-429/200a/b and miRNA-141/200c clusters, as well as miR-431, have also been identified as biomarkers of NEC through microarray analysis [8, 15]. At present, the process of identification of aberrant miRNAs in animals with NEC using NGS technology is still at its nascent stage. This prompted us to identify novel and important miRNAs in animals with NEC in the present study.

The primary objective of this study was to analyze the miRNA expression profiles in rats with NEC. To achieve this, we established an NEC rat model and then sequenced the intestine of these rat pups using NGS technology to identify differentially expressed (DE) miRNAs between the NEC animals and the control animals. We then constructed a DE miRNA and target mRNA regulatory network to recognize the potential functions of these DE miRNAs in NEC.

## 2. Materials and Methods

### 2.1. Ethics Statement

All animal experiments were approved by the Institutional Animal Ethics Committee of Wuxi Maternity and Child Health Care Hospital and carried out in compliance with national and international guidelines for the Care and Use of Laboratory Animals. Major efforts were made to minimize the suffering of the Sprague-Dawley rat pups used in this study.

### 2.2. NEC Animal Experiments

The NEC model was developed as described by Wang et al. [16]. On the first day after birth, nine SPF class Sprague-Dawley rat pups were used, including three in the control group and six in the NEC model group. During the model construction, each rat was gavage fed 200 kcal/kg/day formula (15 g SMA Gold plus, 75 mL PetAg Esbilac Puppy Milk Replacer Liquid) divided into 6 doses per day. The experimental rats were injected with 4 *μ*g/g lipopolysaccharide (LPS) after 8 h of birth, once per day. After LPS, animals were subjected to hypoxia and hypothermia twice a day for two consecutive days. Briefly, animals were exposed to hypoxia in a Plexiglass chamber infused with 100% nitrogen for 60 s, immediately followed by exposure to 4°C for 10 min. The severity of NEC in the rats was assessed based on the NEC scoring system [17]. Grade 0 presented intact morphology (normal), grade 1 displayed sloughing of epithelial cells at the tips of the villi (mild NEC), grade 2 presented midvillous necrosis, grade 3 displayed loss of villi or complete villous necrosis, and grade 4 presented complete destruction of the mucosa, transmural necrosis, and pneumatosis in testinalis. Grade ≥ 2 was considered representative of NEC. There were no deaths during the experimental period. Finally, three NEC model rats were confirmed to be successful according to the pathological results.

### 2.3. Hematoxylin-Eosin (H&E) Staining

The 1.0 cm sections of ileocecum tissues were fixed in 4% paraformaldehyde and then embedded in paraffin. Hematoxylin and eosin (H&E) staining was performed on 4 *μ*m thick sections. After H&E staining, a histopathological examination was performed, and the histopathological condition of the intestine tissues of rats in each group was observed and documented with photographs.

### 2.4. Analysis of Inflammatory Cytokines

The mRNA levels of TNF-*α* and IL-1*β* in the control and NEC groups were measured using real-time quantitative PCR (RT-qPCR). TRIzol reagent (Invitrogen, CA, USA) was utilized to extract the total RNA, according to the manufacturer's protocol. The total RNA was reverse-transcribed with a commercial reverse transcription kit (Thermo Fisher, Waltham, MA, USA), followed by RT-qPCR (2x Master Mix kit from Roche), according to the manufacturer's instructions. FastStart Universal SYBR Green Master mix was used to amplify cDNA on the QuantStudio 6 Flex Real-Time PCR System (Thermo Fisher, Shanghai, China).

### 2.5. Small RNA Library Construction and Sequencing

The small RNA library was constructed using the Multiplex Small RNA Library Prep Kit for Illumina method (NEB, USA), according to the manufacturer's instructions. Following RNA quality control, index codes were added to attribute sequences to each sample. Next, serial processes including 3′ adaptor ligation reaction, RT primer hybridization, 5′ adaptor ligation reaction, and double-stranded cDNA synthesis were performed. The RNA samples were sequenced by Shanghai Yingbai Biotechnology Co. Ltd. using the HiSeq 2500 platform.

### 2.6. miRNA Analysis and DE miRNA Identification

The raw sequences were filtered and optimized to remove short (<15 nt) and low-quality reads. The clean reads were then matched to the miRBase database (http://www.mirbase.org/) to identify known miRNAs (20-25 bp). The raw counts were used as input for the EBSeq algorithm, and DE miRNAs were identified following the criterion (false discovery rate (FDR) < 0.05); FDR was calculated to correct the *p* value.

### 2.7. Target Prediction for the DE miRNAs

The miRanda algorithm (http://www.microrna.org/microrna/home.do) was employed to predict target mRNAs of the DE miRNAs (score ≥ 150 and energy < −20). Subsequently, the RNAhybrid algorithm (https://bibiserv.cebitec.uni-bielefeld.de/RNAhybrid/) was also applied to predict the target mRNAs of the DE miRNAs (energy < −25). The intersection of the two algorithms was taken as the final result.

### 2.8. GO and KEGG Pathway Enrichment Analyses

The GO and KEGG databases were utilized to analyze targeted mRNAs of the DE miRNAs, aimed at the main functions and pathways, respectively. The Fisher test, based on hypergeometric distribution, was used to calculate the *p* value. The results of multiple hypothesis tests were corrected to obtain FDR.

### 2.9. Generation of DE miRNA/mRNA Network

To identify candidate miRNA/mRNA interactions, Cytoscape 2.8.3 was used to establish the regulatory network of DE miRNAs targeting mRNA. The five candidate DE miRNAs and their target mRNAs were imported into Cytoscape to generate and visualize functional networks.

### 2.10. Verification of miRNA Expression by Real-Time Quantitative PCR (RT-qPCR)

The intestinal tissue of the NEC or the control rat was used to extract total RNA using the TRIzol reagent (Invitrogen, CA, USA) and subjected to reverse transcription by PrimeScript™ RT reagent kit (Takara, Dalian, China). RT-qPCR was carried out using the ABI Q6 detection system (Applied Biosystems Inc., MA, USA). GAPDH was used as the endogenous control. The 2^-*ΔΔ*Ct^ method was applied to calculate the relative mRNA expression. Each experiment was performed three times. The primer sequences are given in Table 1.

### 2.11. Statistical Analysis

Data from three independent experiments was expressed as mean ± SD. The *p* values were calculated using GraphPad Prism 8. Student's *t*-test was used for comparisons among groups. *p* < 0.05 was considered statistically significant.

## 3. Results

### 3.1. The Evaluation of Intestinal Injury in Rats with NEC

**Figure 1:**
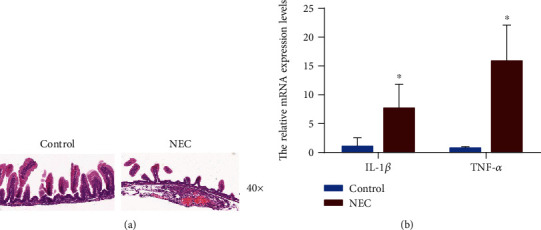
Evaluation of intestinal injury in rats with necrotizing enterocolitis (NEC). (a) Representative electron microscopy images of the ileocecum in rats with or without NEC (40x). Control: *n* = 3; NEC: *n* = 3. (b) RT-qPCR assays showed that TNF-*α* and IL-1*β* levels were significantly higher in the ileocecum tissues of NEC rats than the control rats. The results are expressed as mean ± SD. ^∗^*p* < 0.05.

Pathological changes in intestinal architecture were assessed according to the NEC scoring system [17]. H&E staining of the ileocecum revealed that the control group had no abnormal histological changes; the epithelium was intact and continuous; the glands were arranged neatly; the villi were very high; and there was no hyperemia and edema in the mucosa, submucosa, and lamina propria. However, the NEC group presented severe intestinal tissue necrosis, villi edema, uneven or partial necrosis or disappearance, and severe edema of the lamina propria and submucosa. Thus, the control group presented a grade 0 (normal) degree of injury, and the NEC group presented a degree of injury that was more than grade 2 (Figure 1(a)). Moreover, as indicated by the RT-qPCR result, the mRNA levels of the inflammatory cytokines, TNF-*α* and IL-1*β*, were found to be higher in the NEC group than in the control group (Figure 1(b)). These results confirmed that the NEC model was successfully established.

### 3.2. Changes in the Expression Profiles of miRNAs in NEC

**Figure 2:**
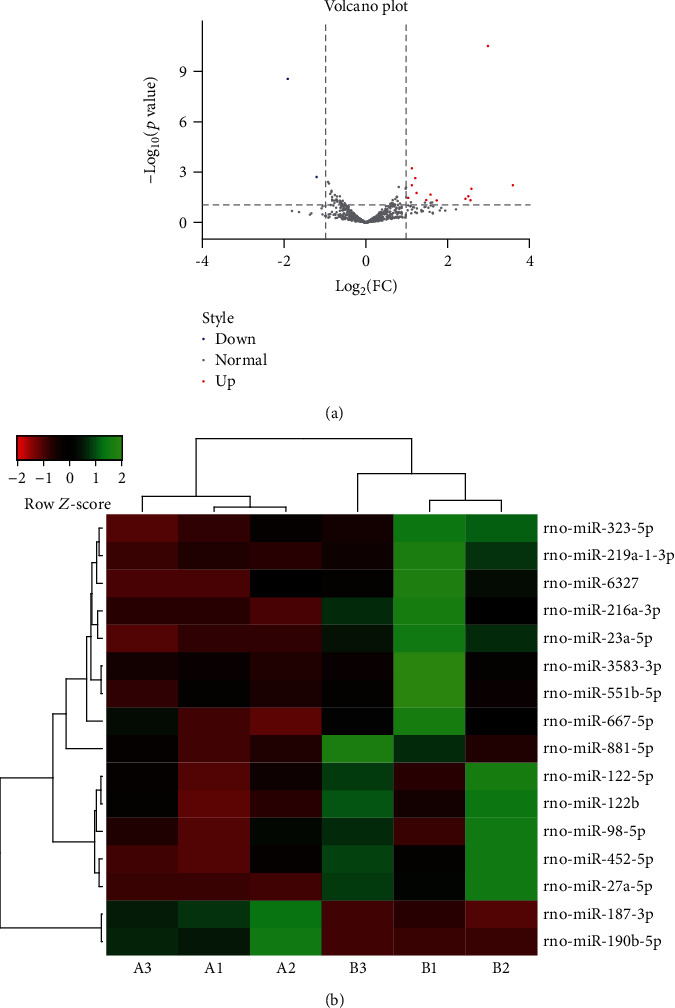
Differentially expressed (DE) miRNAs in the small intestine of rats with or without NEC. (a) The volcano plot shows the DE miRNAs between the NEC rats and the control rats. Red dots and blue dots represent the upregulated and downregulated miRNAs, respectively. The log2^(fold change)^ was ≥1 and FDR was ≤0.05. (b) Heat map of the DE miRNAs in rats with or without NEC. A1, A2, and A3 were the control group; B1, B2, and B3 were the NEC group.

To identify the miRNAs that are differentially expressed between the NEC tissues and the control tissues, we performed miRNA sequencing. After removing low-quality reads from raw reads, an average of 12,214,499 and 13,555,697 clean reads was obtained in the control and NEC groups, respectively, Approximately 97% and 89% of the clean reads were mapped to the genome and Rfam databases, respectively. An overview of the miRNA sequencing data is presented in Table 2. A total of 642 miRNAs were found in the two groups. From these, 16 DE miRNAs were further filtered and identified by using the volcano plot method. Among them, 14 miRNAs were upregulated and only 2 miRNAs were downregulated in the NEC tissues compared to the control tissues (Figure 2(a)). On using the heat map, which is based on a hierarchical cluster, more upregulated DE miRNAs were detected in the NEC group compared to the control group. Thus, the heat map also indicated similar spectral clustering and samples in each group (Figure 2(b)).

### 3.3. Target Gene Prediction, Functional Enrichment, and Pathway Analysis

**Figure 3:**
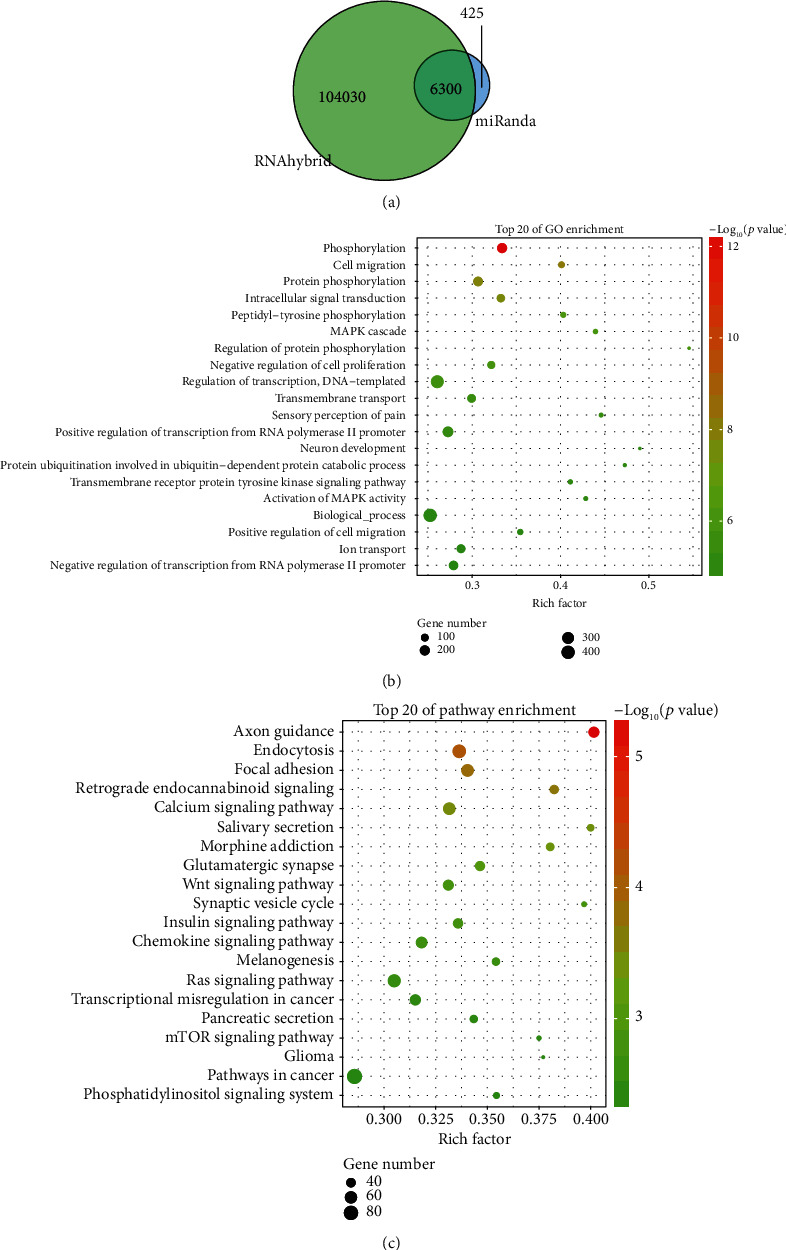
Target gene prediction and functional enrichment analyses of the DE miRNAs. (a) The Venn diagram shows the overlapping predicted target genes for DE miRNAs between the control group and the NEC group using miRanda and RNAhybrid algorithms. (b) Gene Ontology (GO) functional enrichment analysis for the predicted target genes of the DE miRNAs. The *x*-axis shows the enrichment factor including gene numbers and -log_10_(*p* value), and the *y*-axis represents the top 20 GO enrichment terms. (c) Kyoto Encyclopedia of Genes and Genomes (KEGG) functional enrichment analysis for the predicted target genes of the DE miRNAs. The horizontal axis refers to the number of genes and the vertical axis refers to the KEGG pathway terms. Node color: *p* value.

To understand the functions and mechanisms of action of the DE miRNAs, we performed target gene prediction and GO and KEGG pathway analyses. The miRanda and RNAhybrid algorithms identified a total of 6300 target mRNAs for the DE miRNAs between the NEC group and the control group (Figure 3(a)). The top 20 GO terms included some crucial biological processes, such as “phosphorylation,” “cell migration,” and “protein phosphorylation” (Figure 3(b)). The KEGG pathway analysis, on the other hand, provided information concerning gene function. The most enriched pathways included axon guidance, endocytosis, and ribosome and focal adhesion (Figure 3(c)). Among these, the Wnt signaling pathway was the most closely related to NEC.

### 3.4. RT-qPCR Validation of the Candidate miRNAs

**Figure 4:**
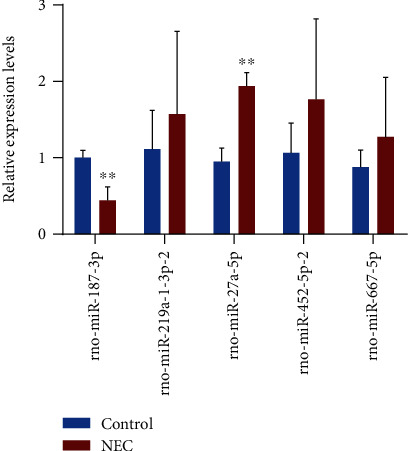
Validation of candidate miRNA expression. The relative expression levels of the five candidate miRNAs were measured by RT-qPCR. The values are expressed as mean ± SD (*n* = 3). ^∗^*p* < 0.05, ^∗∗^*p* < 0.01.

To screen the key miRNAs related to NEC, miRNAs with high fold change and high abundance were selected for further study. Conclusively, the expression levels of the 4 upregulated miRNAs (rno-miR-27a-5p, rno-miR-219a-1-3p, rno-miR-452-5p, and rno-miR-667-5p) and the 1 downregulated miRNA (rno-miR-187-3p) were verified by RT-qPCR. Thus, the expression patterns of these five DE miRNAs, as analyzed by RT-qPCR, were in accordance with the expression patterns obtained by miRNA sequencing. However, only the expression patterns of rno-miR-27a-5p and rno-miR-187-3p were found to be statistically significant (Figure 4).

### 3.5. miRNA/mRNA Interaction Network Analysis

**Figure 5:**
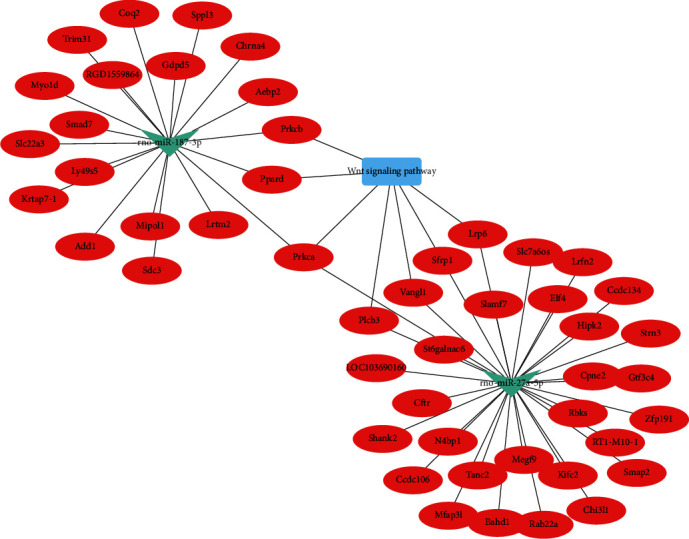
The DE miRNA/mRNA interaction network constructed and visualized using Cytoscape software. Integrated miRNA/mRNA/pathway analysis of potential target mRNAs associated with the upregulated miRNA, rno-miR-27a-5p, and the downregulated miRNA, rno-miR-187-3p. The predicted target mRNAs of these two miRNAs were found to be enriched in the Wnt signaling pathway. *p* < 0.01. The triangle represents the DE miRNAs. The oval represents the target mRNAs. The rectangle represents the Wnt signaling pathway.

We used Cytoscape software to depict an integrated mRNA/miRNA interaction network that included the upregulated miRNA, rno-miR-27a-5p, and the downregulated miRNA, rno-miR-187-3p. Bioinformatics analysis showed that the target mRNAs of these two miRNAs were enriched in the Wnt signaling pathway. In this complex network, the target genes for rno-miR-27a-5p were Prkca, Plcb3, Vangl1, and Sfrp1, while the target genes for rno-miR-187-3p were Prkca, Ppar, and Prkcb (Figure 5).

## 4. Discussion

NEC is a high mortality disease that is responsible for 1–3 deaths per 1000 births per year among newborn infants in North America [18, 19]. To date, little is known about the role of the miRNAs in premature infants with NEC. In our study, we constructed an NEC rat model and obtained the expression profiles of miRNAs in this model. We also identified 16 differentially expressed (DE) miRNAs between the NEC group and the control group. Furthermore, the top 20 enriched GO terms and pathways were identified. Five DE miRNAs were then further screened and verified by RT-qPCR, the results for which were in accordance with the results of miRNA-seq. In addition, we used bioinformatics analysis to predict the miRNA/mRNA interactions. These findings indicate that the identified DE miRNAs target genes that might participate in phosphorylation processes and the Wnt signaling pathway in NEC. Thus, the identified DE miRNAs hold promise as potential therapeutic targets.

In 2008, the first study to report miRNA expression patterns using tissue samples from inflammatory bowel disease patients was published [20]. miRNAs play a vital role in the differentiation and function of the intestinal epithelium [21]. A study reports that miR-31 can reduce the inflammatory response in the colon epithelium of mice and also regulate the Wnt signaling pathway to promote epithelial regeneration, thus reducing the features of colitis [22]. Knowledge about the mechanism of action and functional role of miRNAs in both noninflammatory neonatal and NEC tissues is very limited. Additionally, it has been reported that Wnt5a can promote interferon-*γ* signaling, leading to IL-12 expression in dendritic cells, thereby inducing Th1 differentiation in colitis [23]. It is speculated that genetic variants of SMAD2/3/4/7 might alter the balance of differentiation between Th17 and T, resulting in the development of inflammatory bowel disease, including ulcerative colitis [24]. Smad3-dependent disruption of the TGF-*β* signaling pathway has been shown to impair the healing of murine intestinal mucosal ulcers [25]. In the present study, the expression levels of rno-miR-27a-5p and rno-miR-187-3p were shown to be significantly different in the NEC group compared to the control group. Moreover, the expression patterns of rno-miR-219a-1-3p, rno-miR-452-5p, and rno-miR-667-5p, as measured by RT-qPCR, were in accordance with those obtained by miRNA-seq. The predicted target genes of these miRNAs are related to intestinal injury and colitis; thus, their regulation may affect the development of NEC.

The pathogenesis of NEC is characterized by intestinal inflammation and injury. Previous reports state that the Wnt/*β*-catenin signaling pathway is essential for intestinal renewal, deregulation of which results in impairment of intestinal epithelial stem cell proliferation and differentiation [26]. For instance, inhibition of Wnt/*β*-catenin signaling leads to defective intestinal regeneration in mice and humans with NEC. Exogenously administered Wnt7b has the potential to maintain intestinal epithelial homeostasis and avoid NEC intestinal injury [27]. In the present study, the target genes of the DE miRNAs were found to participate in the Wnt signaling pathway. Thus, our results provide additional evidence that the abnormal Wnt signaling pathway plays an important role in the pathogenesis of NEC. Immune cells control epithelial stem cells through inflammatory cytokines, either directly or by regulating Wnt signaling [28]. The proinflammatory cytokines, TNF-*α* and IFN-*γ*, can synergistically drive epithelial barrier dysfunction and apoptosis, particularly during colitis [29]. Wnt5A induces special inflammatory cytokines that partly cooperate to identify receptor signaling [30, 31]. In our study, the expression levels of TNF-*α* and IL-1*β* were found to be higher in the NEC group than in the control group. These results reveal that in rats with NEC, the occurrence of intestinal inflammation may be regulated by Wnt signaling.

## 5. Conclusion

In conclusion, we identified 16 DE miRNAs between the NEC group and the control group. The target genes of the significant DE miRNAs were found to be enriched in the Wnt signaling pathway. We hypothesize that the identified miRNAs might be potentially involved in inflammatory response through the Wnt signaling pathway. In our future studies, we wish to further explore and verify this hypothesis.

## Figures and Tables

**Table 1:** Primer sequences for real-time quantitative PCR.

Primer name	Sequence (5′-3′)
rno-miR-27a-5p	GGGCTTAGCTGCTTGTG
rno-miR-187-3p	GCAGTCGTGTCTTGTGTTG
rno-miR-219a-1-3p	GAGAGTTGCGTCTGGAC
rno-miR-667-5p	GCTGGTGGAGCAGTGAG
rno-miR-452-5p	CAGAACTGTTTGCAGAGGA
All-R	AGTGCGTGTCGTGGAGTCG

**Table 2:** Summary of miRNA sequencing datasets.

Sample name	Total reads	Clean reads	GC (%)	Mapped reads (reference genome)	Mapped reads (Rfam database)
A1	12,574,251	11,549,212	48	5,901,044 (97%)	10,099,878 (88%)
A2	14,119,049	13,030,020	48	8,544,838 (97%)	11,520,278 (88%)
A3	15,991,788	12,064,266	49	8,134,694 (97%)	10,916,943 (91%)
B1	13,571,708	12,596,388	49	7,474,852 (98%)	10,713,059 (85%)
B2	14,749,506	13,805,180	49	9,330,200 (97%)	12,687,170 (92%)
B3	15,395,087	14,265,523	50	10,416,571 (97%)	12,936,944 (91%)

## Data Availability

The datasets used and/or analyzed during the current study are available from the corresponding author on reasonable request.
